# Supplementary figures and images for: Comparative analysis of rhizosphere soil between three plantation types in Karst Rocky Desertification area by widely targeted metabolomics
Source: PeerJ. 2025 Mar 31;13:e19131. doi: 10.7717/peerj.19131 (PMC11967436; doi:10.7717/peerj.19131)

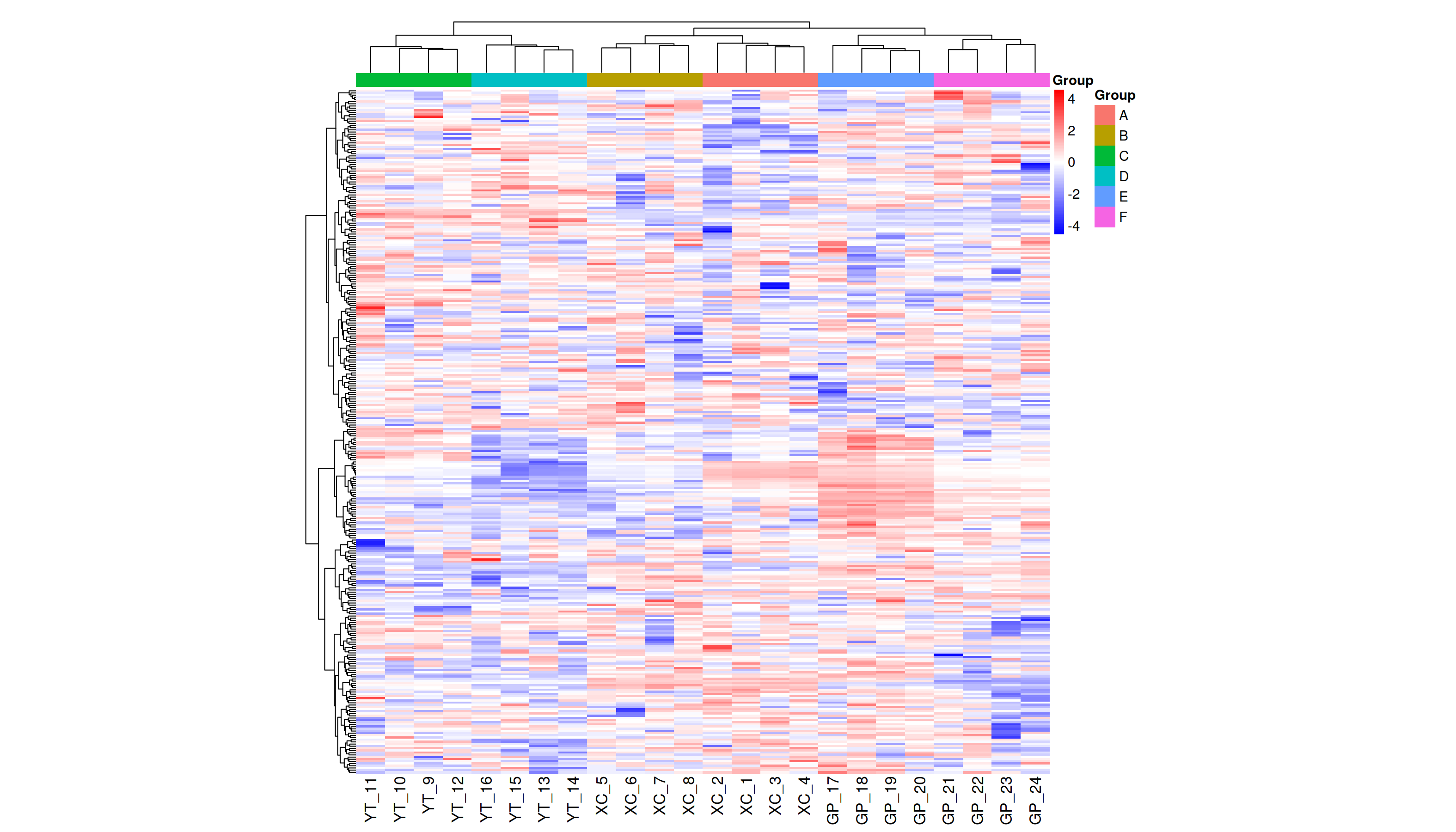

Supplement: Supplemental Information 1 [file peerj-13-19131-s001.png]

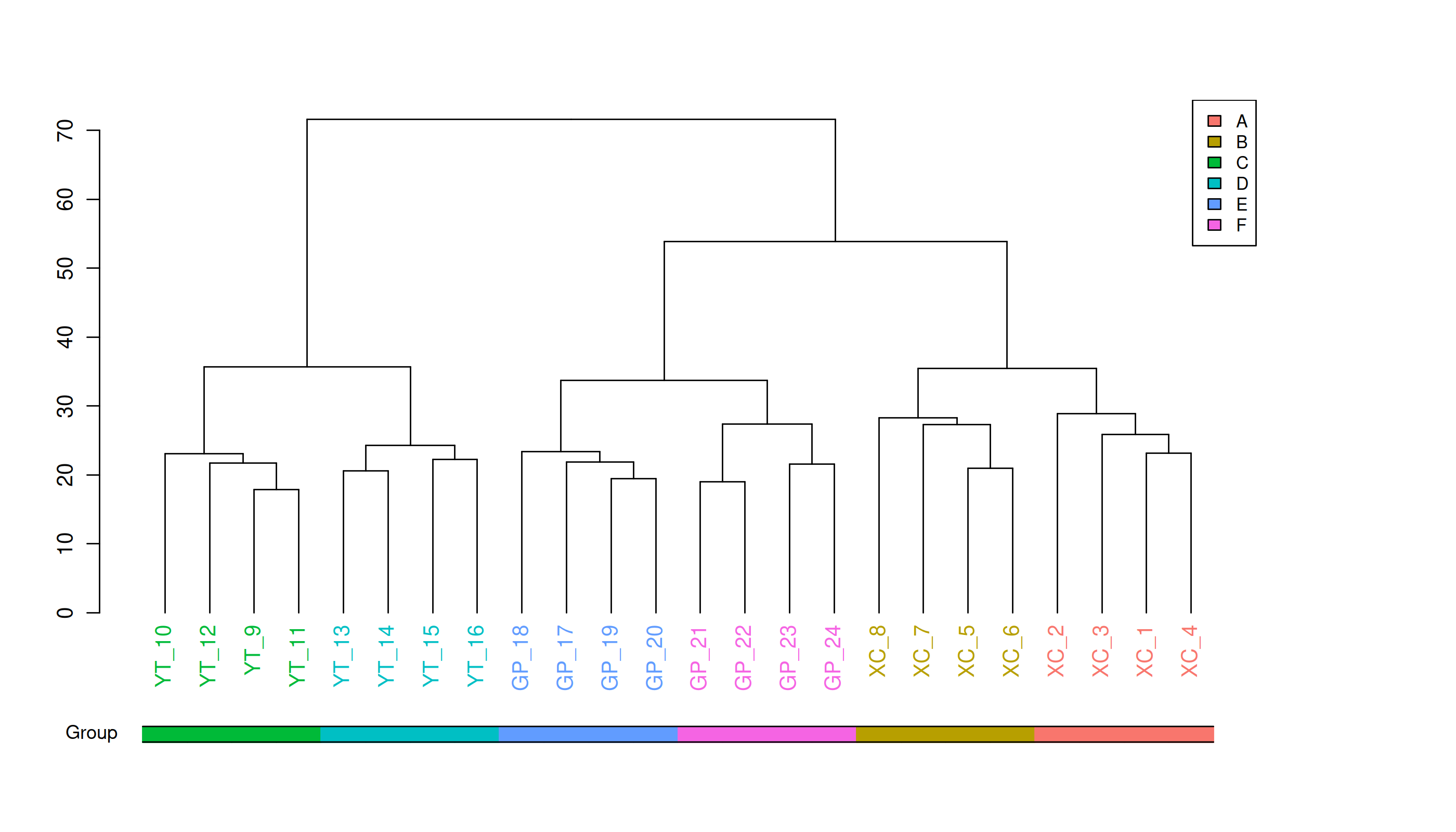

Supplement: Supplemental Information 2 [file peerj-13-19131-s002.png]

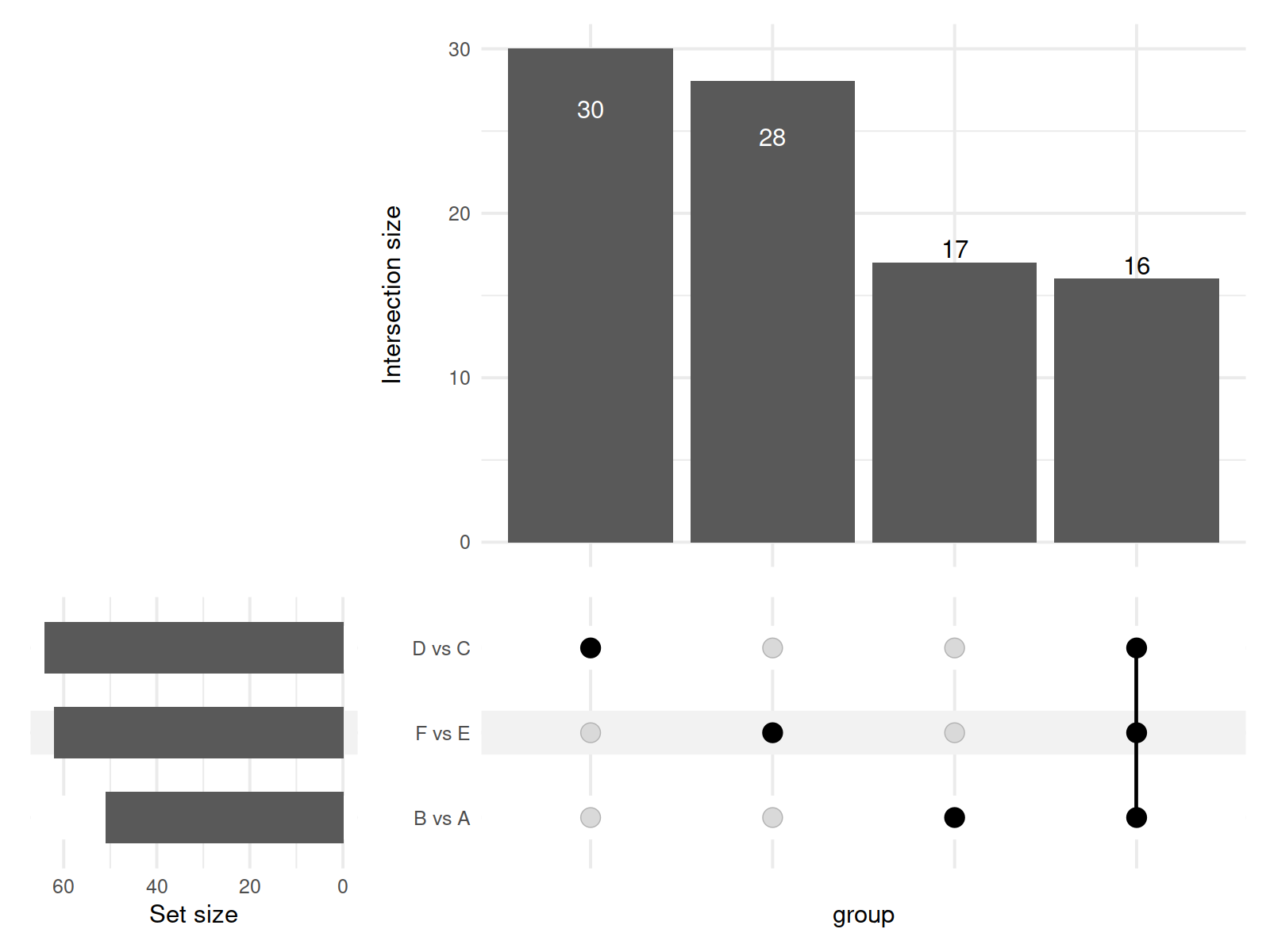

Supplement: Supplemental Information 3 [file peerj-13-19131-s003.png]

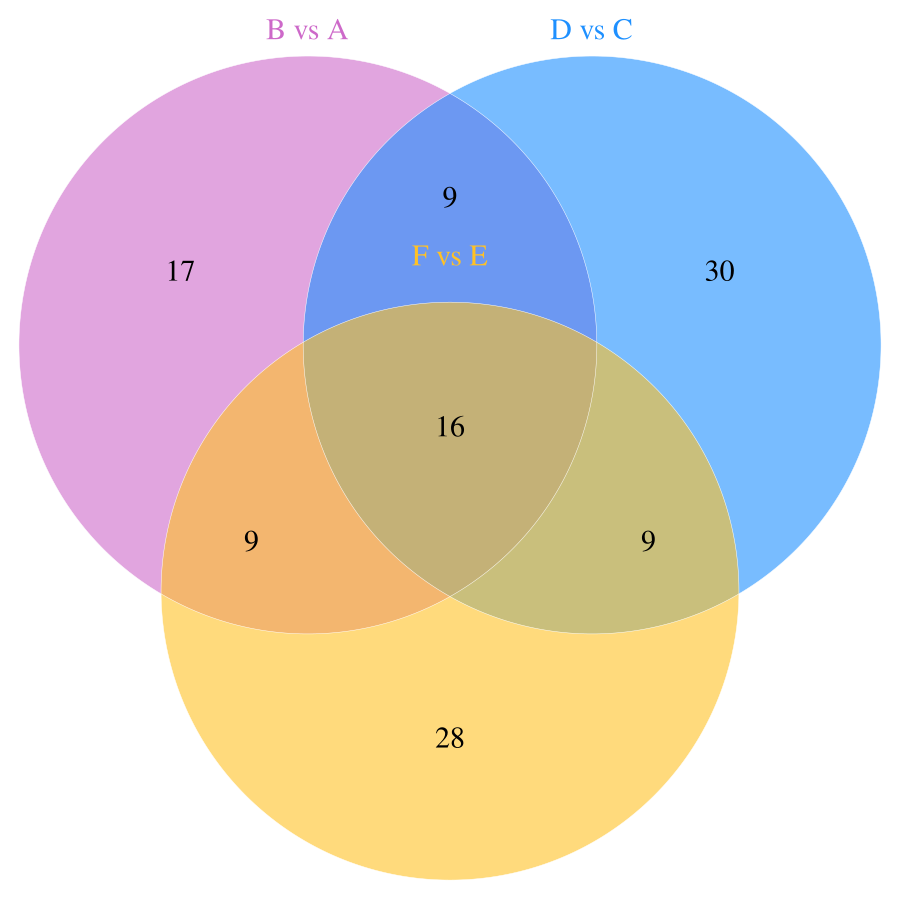

Supplement: Supplemental Information 4 [file peerj-13-19131-s004.png]

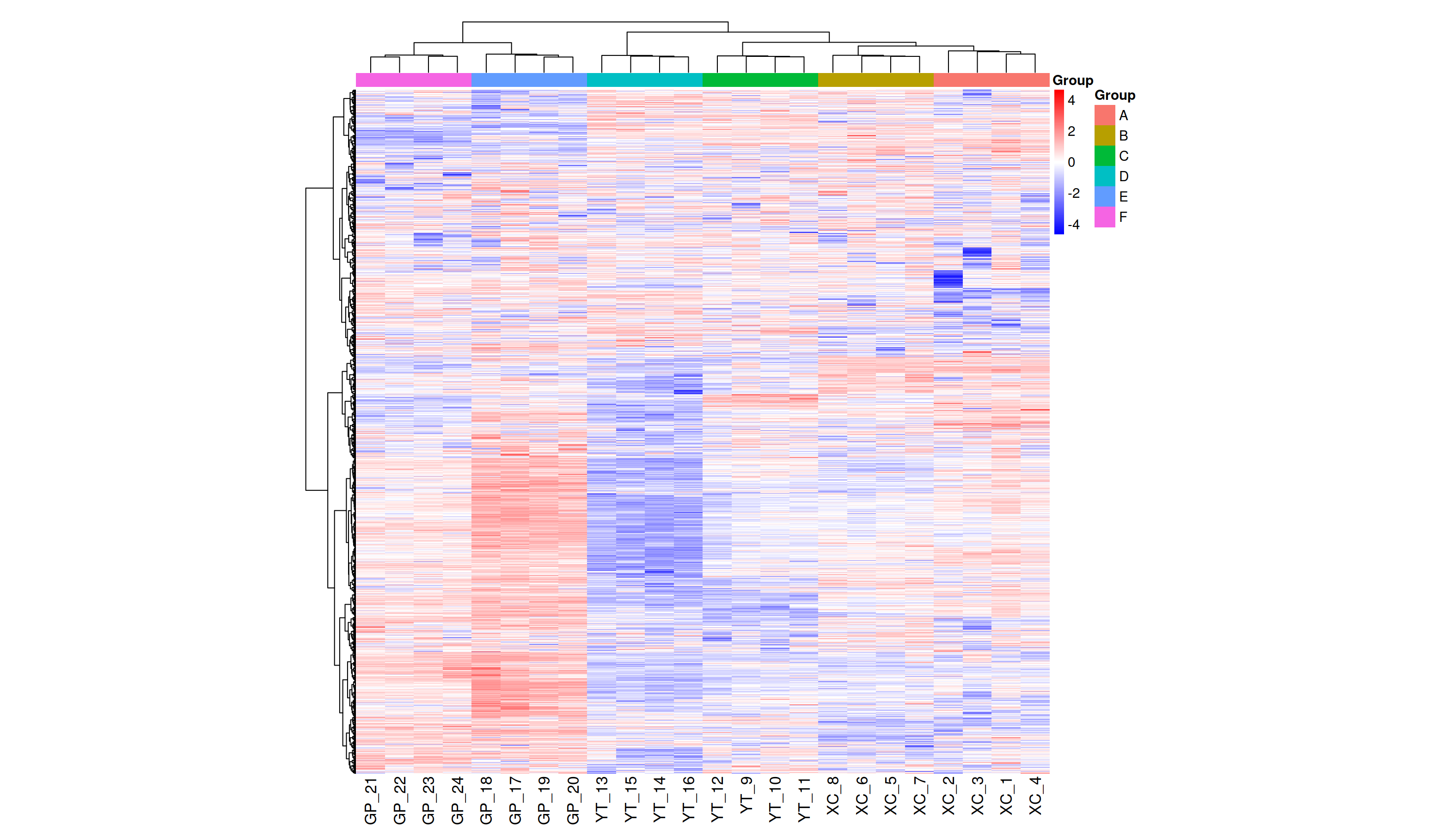

Supplement: Supplemental Information 5 [file peerj-13-19131-s005.png]

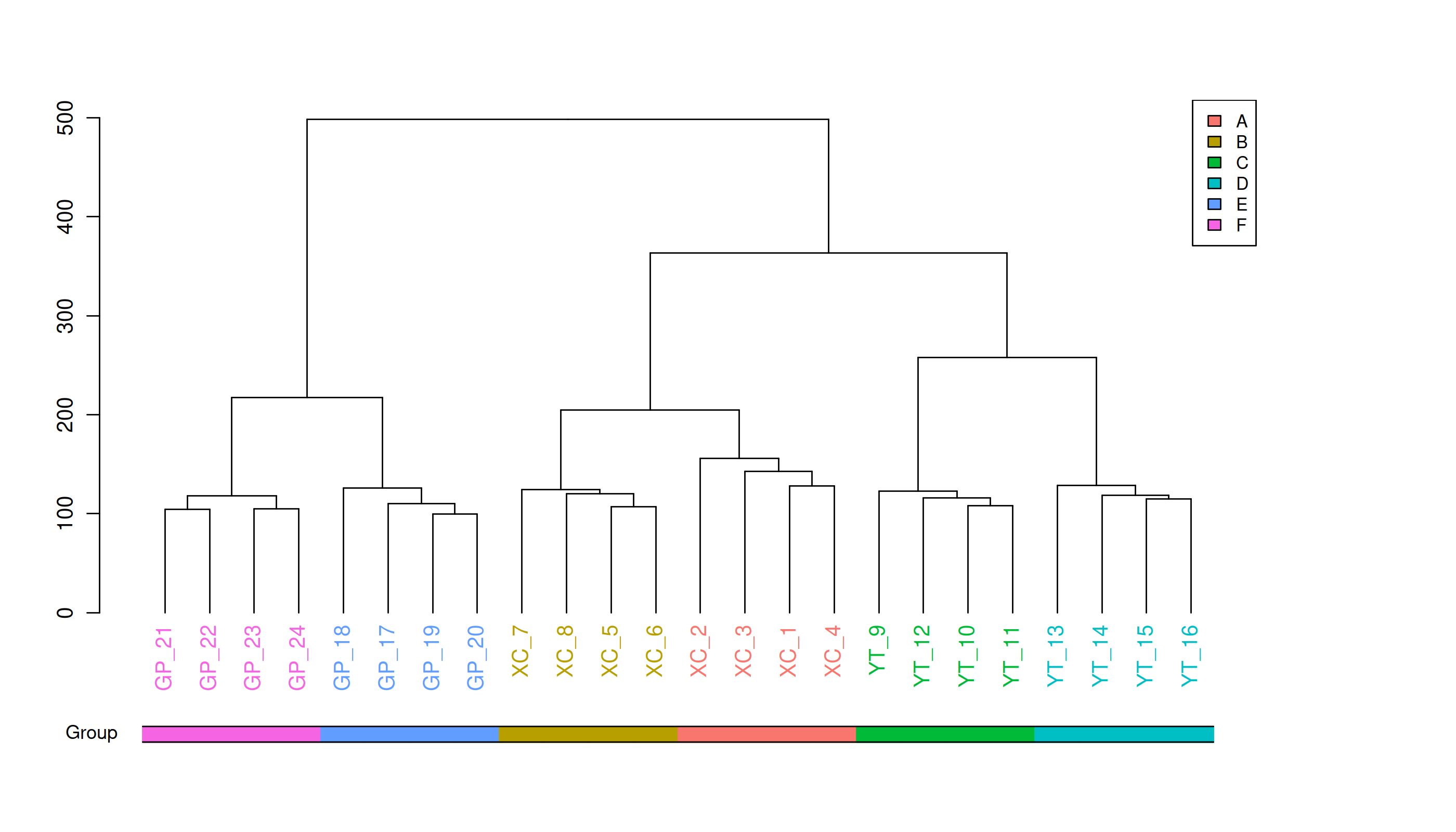

Supplement: Supplemental Information 6 [file peerj-13-19131-s006.png]

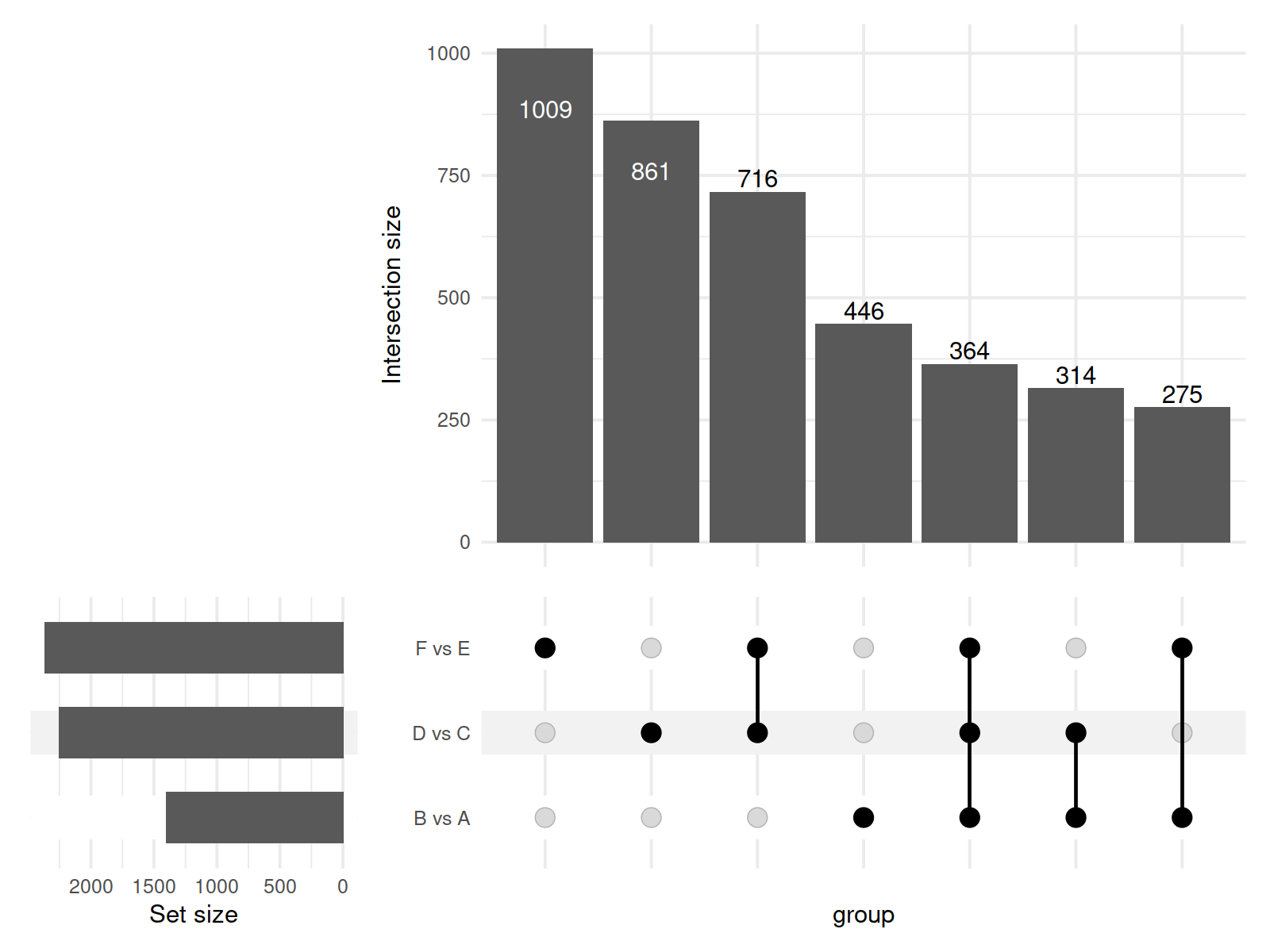

Supplement: Supplemental Information 7 [file peerj-13-19131-s007.png]

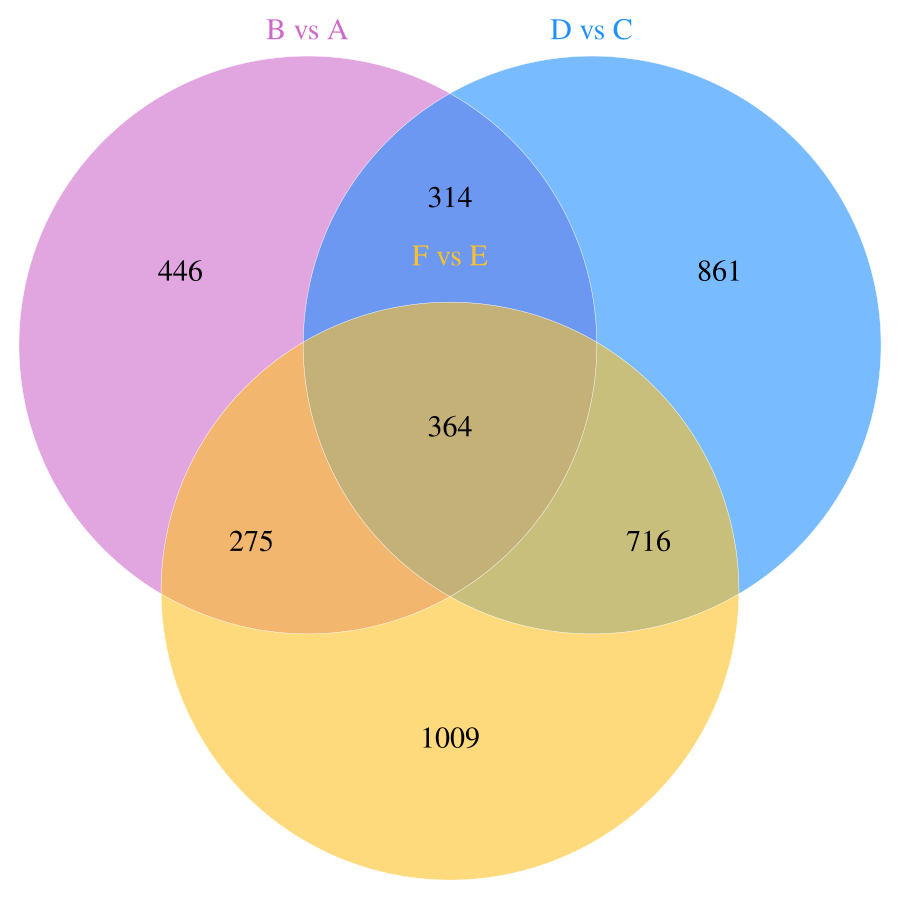

Supplement: Supplemental Information 8 [file peerj-13-19131-s008.png]

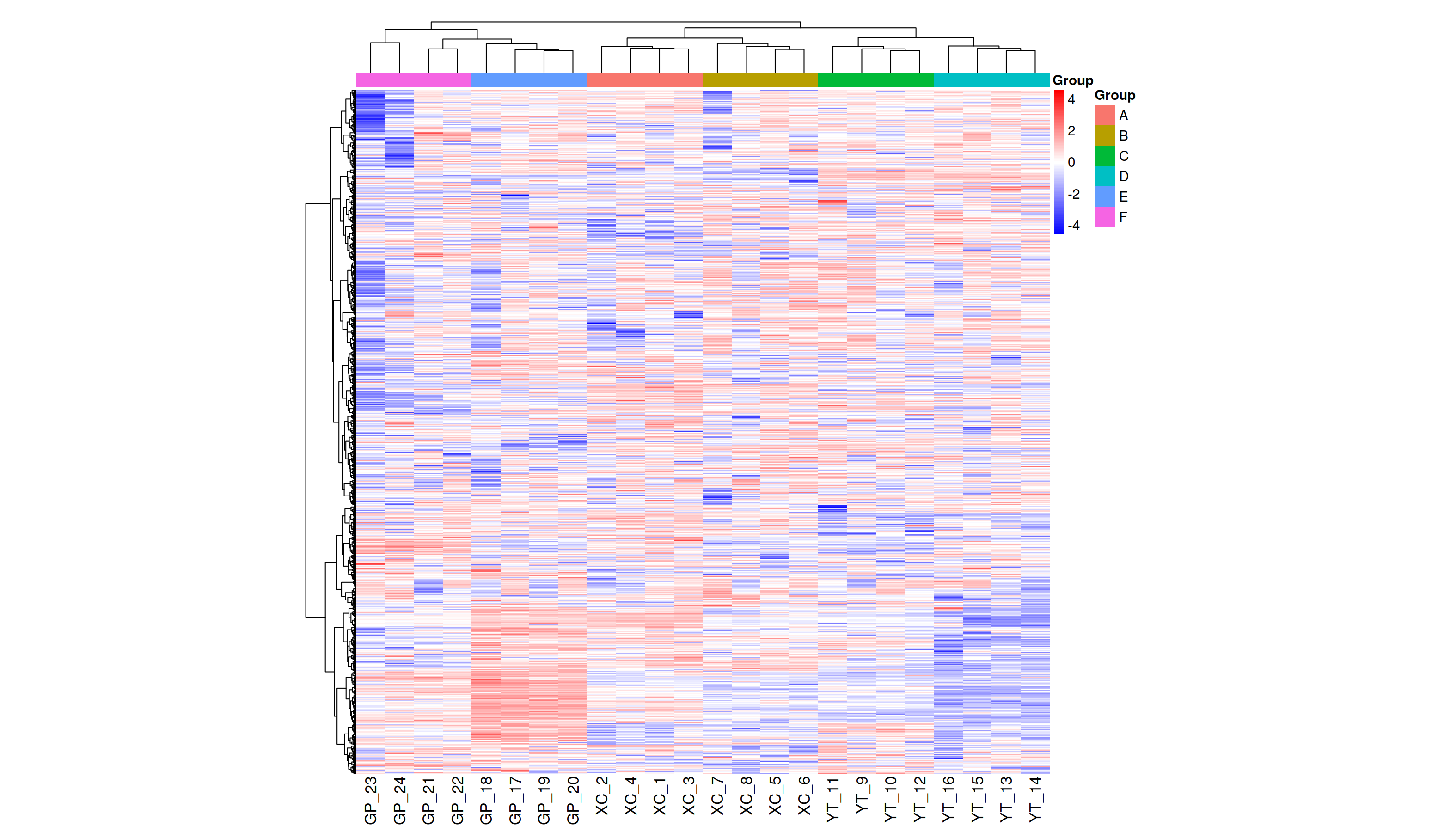

Supplement: Supplemental Information 9 [file peerj-13-19131-s009.png]

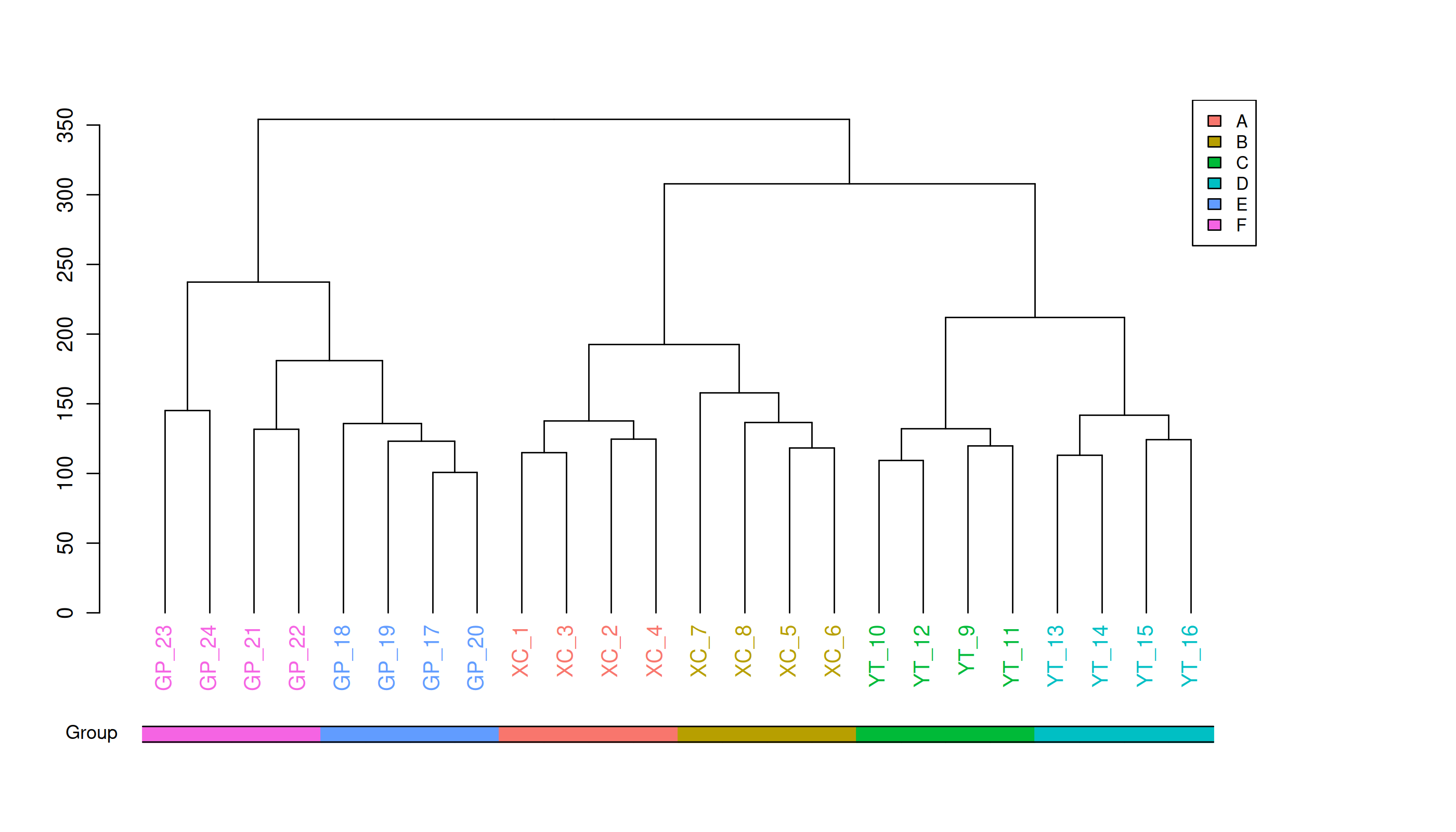

Supplement: Supplemental Information 10 [file peerj-13-19131-s010.png]

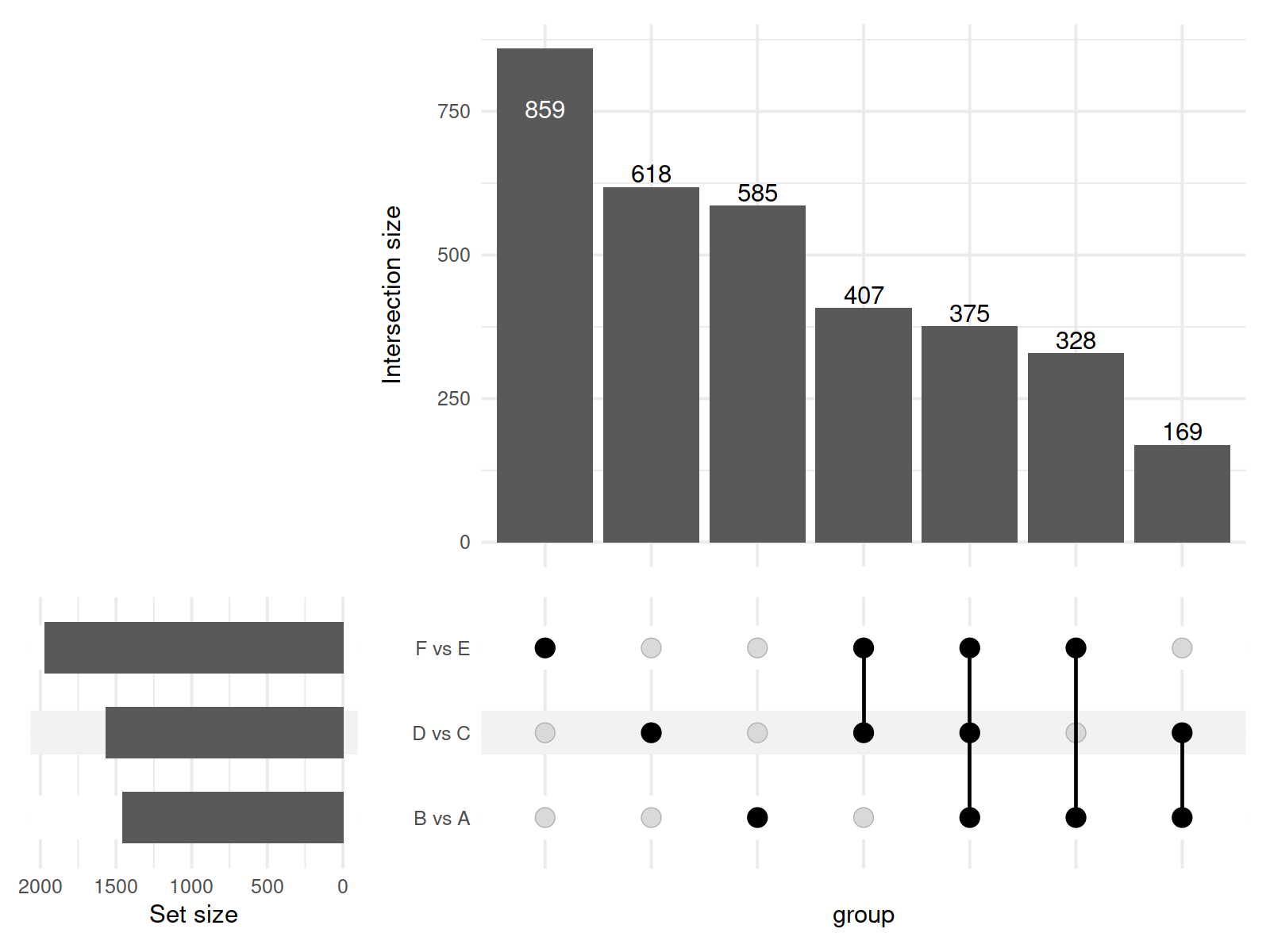

Supplement: Supplemental Information 11 [file peerj-13-19131-s011.png]

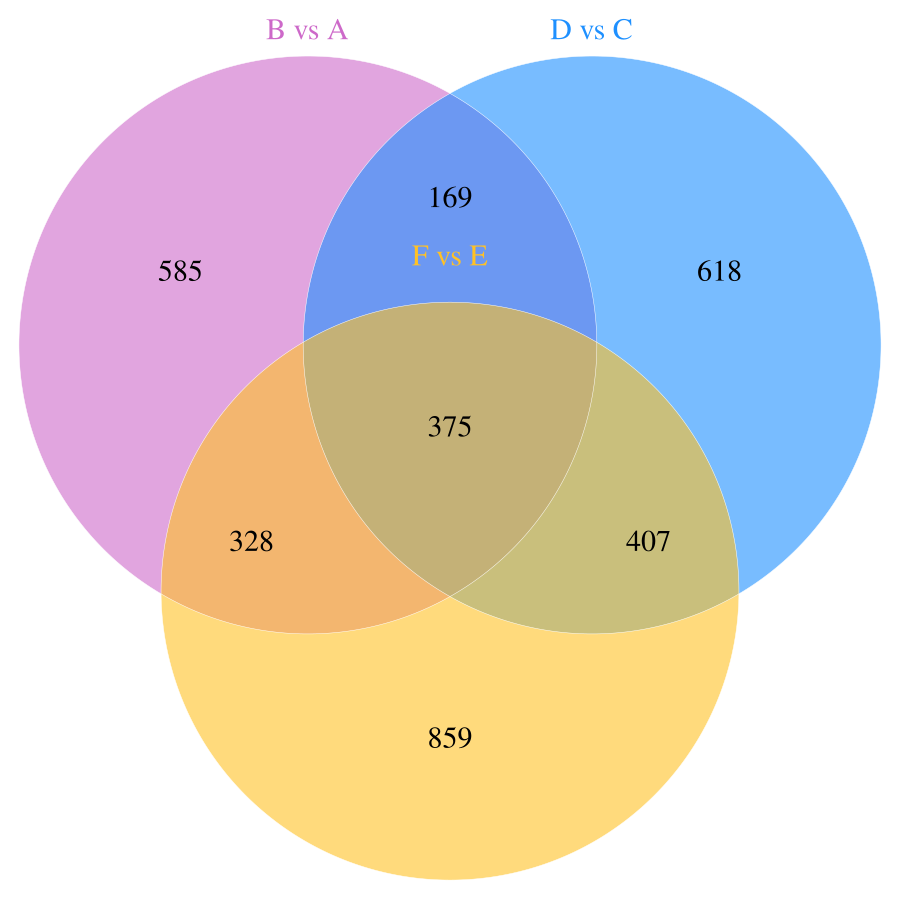

Supplement: Supplemental Information 12 [file peerj-13-19131-s012.png]
